# A Boolean Model of the Gene Regulatory Network Underlying Mammalian Cortical Area Development

**DOI:** 10.1371/journal.pcbi.1000936

**Published:** 2010-09-16

**Authors:** Clare E. Giacomantonio, Geoffrey J. Goodhill

**Affiliations:** 1 Queensland Brain Institute, The University of Queensland, St Lucia, Queensland, Australia; 2 School of Mathematics and Physics, The University of Queensland, St Lucia, Queensland, Australia; University College London, United Kingdom

## Abstract

The cerebral cortex is divided into many functionally distinct areas. The emergence of these areas during neural development is dependent on the expression patterns of several genes. Along the anterior-posterior axis, gradients of Fgf8, Emx2, Pax6, Coup-tfi, and Sp8 play a particularly strong role in specifying areal identity. However, our understanding of the regulatory interactions between these genes that lead to their confinement to particular spatial patterns is currently qualitative and incomplete. We therefore used a computational model of the interactions between these five genes to determine which interactions, and combinations of interactions, occur in networks that reproduce the anterior-posterior expression patterns observed experimentally. The model treats expression levels as Boolean, reflecting the qualitative nature of the expression data currently available. We simulated gene expression patterns created by all 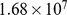 possible networks containing the five genes of interest. We found that only 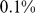 of these networks were able to reproduce the experimentally observed expression patterns. These networks all lacked certain interactions and combinations of interactions including auto-regulation and inductive loops. Many higher order combinations of interactions also never appeared in networks that satisfied our criteria for good performance. While there was remarkable diversity in the structure of the networks that perform well, an analysis of the probability of each interaction gave an indication of which interactions are most likely to be present in the gene network regulating cortical area development. We found that in general, repressive interactions are much more likely than inductive ones, but that mutually repressive loops are not critical for correct network functioning. Overall, our model illuminates the design principles of the gene network regulating cortical area development, and makes novel predictions that can be tested experimentally.

## Author Summary

Understanding the development of the brain is an important challenge. Progress on this problem will give insight into how the brain works and what can go wrong to cause developmental disorders like autism and learning disability. This paper examines the development of the outer part of the mammalian brain, the cerebral cortex. This part of the brain contains different areas with specialised functions. Over the past decade, several genes have been identified that play a major role in the development of cortical areas. During development, these genes are expressed in different patterns across the surface of the cortex. Experiments have shown that these genes interact with each other so that they each regulate how much other genes in the group are expressed. However, the experimental data are consistent with many different regulatory networks. In this study, we use a computational model to systematically screen many possible networks. This allows us to predict which regulatory interactions between these genes are important for the patterns of gene expression in the cortex to develop correctly.

## Introduction

The mammalian cerebral cortex is a complex but extremely precise structure. In adult, it is divided into several functionally distinct areas characterised by different combinations of gene expression, specialised cytoarchitecture and specific patterns of input and output connections. But how does this functional specification arise? There is strong evidence that both genetic and activity-dependent mechanisms play a role in the development of these specialised areas, a process also referred to as arealisation. A genetic component is implicated by the spatial non-uniformity of expression of some genes prior to thalamocortical innervation, as well as the fact that altering expression of some genes early in development changes area position in adult [for review see 1]–[8]. On the other hand, manipulating thalamocortical inputs, and hence activity from the thalamus, can alter area size or respecify area identity [for review see 1], [4], [8]. These results are accommodated in a current working model of cortical arealisation as a multi-stage process where initial broad spatial patterns of gene expression provide a scaffold for differential thalamocortical innervation [5]. Patterned activity on thalamocortical inputs then drives more complex and spatially restricted gene expression which, in turn, regulates further area specific differentiation. This paper focuses on the earliest stage of arealisation: how patterns of gene expression form early in cortical development.

**Figure 1:**
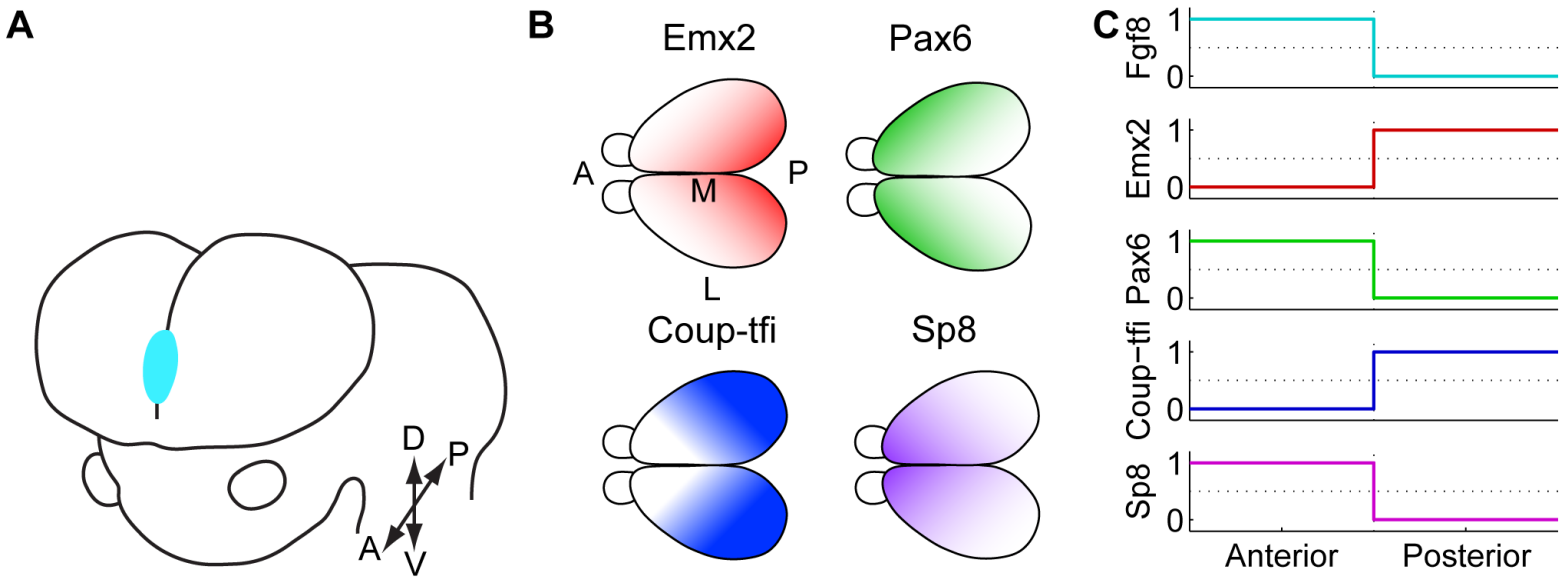
Gene expression in the developing neocortex. (**A**) The anterior neural ridge or commissural plate (*blue*) is a patterning centre in the developing forebrain that secretes the morphogen Fgf8. Since the protein is secreted, it is hypothesised that it diffuses to form a gradient [5]. The directions A, P, D, V, M and L indicate anterior, posterior, dorsal, ventral, medial and lateral respectively. (**B**) These four transcription factors are expressed in spatial mRNA and protein gradients across the developing forebrain. Many other genes with spatial patterns of expression have also been identified [for review see 8]. (**C**) A schematic of the desired steady state expression levels in the anterior and posterior compartments in the discretised Boolean model. A is adapted from Figure 1A in [4] and Figure 1 in [5], B is adapted from Figure 6A in [5].

**Figure 2:**
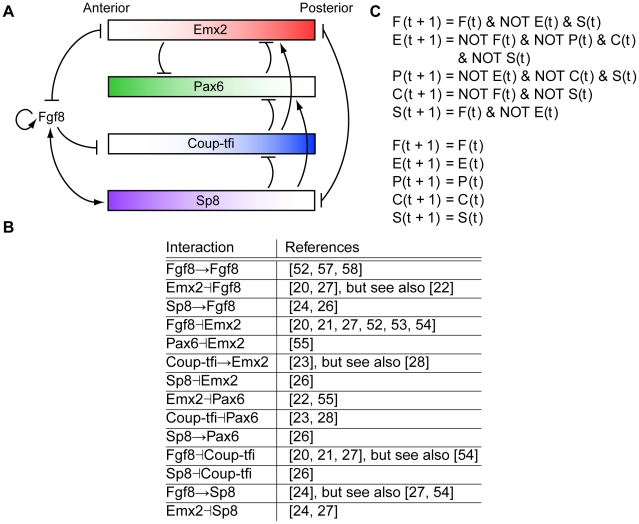
A network created by interactions between the five genes of interest as suggested by experiments. (**A**) Arrows (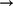) indicate inductive or activating interactions, flat bars (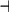) indicate repressive interactions. Text in italics signifies genes while upright text signifies proteins. Only the activation of *Fgf8* by Sp8 (Sp8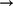 *Fgf8*) has been directly demonstrated [24]. Other interactions have generally been inferred based on altered expression patterns in mutants and therefore might be indirect. For example, the activation of *Emx2* by *Coup-tfi* might be due to *Coup-tfi* repressing *Pax6* which in turn represses *Emx2*. This panel is adapted from Figure 6*B* in [5]. (**B**) References for each of the interactions in panel A. (**C**) The Boolean logic equations for the network in panel A. *F*, *E*, *P*, *C* and *S* are the logical variables representing the genes *Fgf8*, *Emx2*, *Pax6*, *Coup-tfi* and *Sp8* and F, E, P, C and S are the logical variables representing the respective proteins. For a gene to be turned on at time 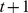, its inductive regulators must be present and its repressive regulators absent at time 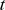.

Experiments have identified many genes expressed embryonically that are critical to the positioning of cortical areas in adult. Although arealisation occurs in a two-dimensional field, most experiments focus on anterior-posterior patterning and hence, here we concentrate on patterning along this axis. From around embryonic day 8 (E8) in mouse, the morphogen *Fgf8* is expressed at the anterior pole of the developing telencephalon (Figure 1A) [2], [3], [5], [7]–[11]. Immediately after *Fgf8* expression is initiated in mouse, four transcription factors (TFs), *Emx2*, *Pax6*, *Coup-tfi* and *Sp8* are expressed in gradients across the surface of the cortex (Figure 1B) [2], [3], [5], [8], [11]. These four TFs are an appealing research target because their complementary expression gradients could provide a unique coordinate system for arealisation [5], equivalent to “positional information” [12], [13]. Altered expression of each of *Fgf8* and the four TFs shifts area positions in late embryonic stages and in adult [14]–[29]; [ but see also 30]. Furthermore, during development, altered expression of each of these genes up- or down-regulates expression of some other genes in the set along the anterior-posterior axis (see Figure 2B for references). A large cohort of experiments has given rise to a hypothesised network of regulatory interactions between these five genes (Figure 2A). However, only one of these interactions has been directly demonstrated [24] and no analysis has been performed at the systems level.

Interacting TFs are known to be able to form regulatory networks that drive differential spatial development, fulfilling a role for which morphogens are better known [31], [32]. Feedback loops are the crucial feature that enable the generation of spatial (and temporal) patterns of expression of the genes in the network. Since TFs regulate the expression of other genes, local differences in expression of a set TFs are a powerful method of generating spatial patterns of growth, differentiation and expression of guidance cues (and therefore innervation), and developing more complex patterns of gene expression. The arealisation genes form a regulatory network with many feedback loops which is in principle capable of generating spatial patterns. Establishing which interactions are critical for correct arealisation is of great interest to the field, but current experimental approaches are limited in their ability to quickly assay the importance of each particular interaction.

Computational modelling of gene regulatory networks is necessary because their complex behaviour is difficult to understand intuitively. In addition, it offers several other benefits. Currently, the many hypothesised interactions between arealisation genes are represented as arrow diagrams like that seen in Figure 2A. Because intuition tends to follow simple causal chains, the presence of many feedback loops makes intuition about the overall behaviour of complex systems unreliable [33]–[37]. Consequently, a more formal description than an arrow diagram would test the current conceptual model, and has the potential to give greater understanding and insight, as it has done for many other regulatory networks [for review see 33]–[36], [38]–[44]. The unambiguous descriptions found in mathematical and computational models offer the added benefit of making assumptions explicit and therefore allowing greater scrutiny [45]. Computational experiments can also be performed quickly and cheaply relative to laboratory experiments and consequently can be useful for conducting thought experiments which can then be tested experimentally [45], [46]. In this way, computational modelling and experiments can spur each other on so that both are “improved in a synergistic manner” [36].

Here, we use the Boolean logical approach to model the arealisation regulatory network. In this approach, variables representing genes and proteins can take only two values, zero or one, representing gene and protein activity being below or above some threshold for an effect. While continuous models are more realistic, they have many free parameters which are hard to constrain from experimental data, and offer a formidable computational challenge to investigate systematically. In contrast, Boolean models can be used when only qualitative expression and interaction data are available, as is the case for arealisation. In Boolean models, at each point in time, the state of a variable depends on the state of its regulators at the previous time step. A set of logic equations capture the regulatory relationships between variables and dictate how the system evolves in time. The Boolean idealisation greatly reduces the number of free parameters while still capturing network dynamics and producing biologically pertinent predictions and insights [43], [45], [47]. In our model, we use only two spatial compartments, one representing the anterior pole and another representing the posterior pole. The anterior and posterior expression levels after Boolean discretisation are shown in Figure 1C. More than two expression levels and more than two spatial compartments would be more realistic, but would result in an explosion in the number of parameters currently unconstrained by experimental data. Having only two expression levels and only two compartments allows us to systematically screen a large number of networks, which would be impossible in a more complex model.

In this paper, we simulate the dynamics of all possible networks created by different combinations in interactions between *Fgf8*, *Emx2*, *Pax6*, *Coup-tfi* and *Sp8*, and show that only 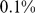 of these networks are able to reproduce the expression patterns observed experimentally. From this analysis, we identify structural elements common to the best performing networks, as well as elements that never appear in the networks that perform well. These results reveal important logical principles underlying the cortical arealisation gene network, and suggest potential directions for future experimental investigations.

## Results

### Simulation of the dynamics of 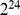 possible networks revealed networks that reliably reproduced the experimentally observed expression gradients

Experimental evidence indicates that *Fgf8*, *Emx2*, *Pax6*, *Coup-tfi* and *Sp8* regulate each other's expression, but the actual structure of the network is highly unconstrained by experimental data. Hence, we performed a systematic screen of the different possible networks and then looked for common structural features in the networks that perform poorly and well.

We analysed the dynamics of all networks created by different combinations of 24 possible interactions between these five genes and their respective proteins. In each network, Sp8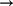 *Fgf8* was fixed since this has been directly demonstrated [24]. We also did not consider positive interactions between species with opposing expression gradients, or negative interactions between species with the same gradient. For example, Emx2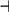 *Pax6* and Fgf8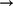 *Pax6* were possible interactions, but Emx2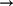 *Pax6* and Fgf8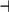 *Pax6* were not. The 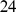 variable interactions generated 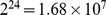 possible networks. The structure of each network was transformed into a set of Boolean logic functions as described in the Methods.

We identified networks that proceeded from the state at the anterior pole at E8 to the state at around E10.5, as well as from the state at the posterior pole at E8 to the state at around E10.5. At E8, of our genes of interest, only *Fgf8* is active due to mechanisms external to the network we are modelling [5], [24], [26], and only at the anterior pole. Hence, in the Boolean model with binary variables, *Fgf8* gene and protein started in the active (‘1’) state in the anterior compartment and inactive (‘0’) state in the posterior compartment, while the other genes and proteins started in the inactive state in both compartments. By E10.5, the expression patterns seen in Figure 1 are present. That is, at the anterior pole, *Fgf8*, *Pax6* and *Sp8* genes are active, while *Emx2* and *Coup-tfi* are inactive; at the posterior pole, *Emx2* and *Coup-tfi* are active, while *Fgf8*, *Pax6* and *Sp8* are inactive.

When the Boolean update functions describing a network were applied stochastically, many networks reached multiple steady states with fixed probabilities. In these cases, we calculated the average gene and protein levels, weighted by the probability of ending in a particular state, and thus each Boolean variable could be between 0 and 1. We say that a network reliably reaches a desired steady state if it does so with a greater than 50% probability. From this, it follows that networks that reliably reach both the anterior and posterior steady states from the respective starting states have differences in activity between the anterior and posterior poles than span 0.5, as in Figure 1C. We define these networks as good.

### A previously hypothesised regulatory network does not satisfy our criteria for reproducing the experimental observations

**Figure 3:**
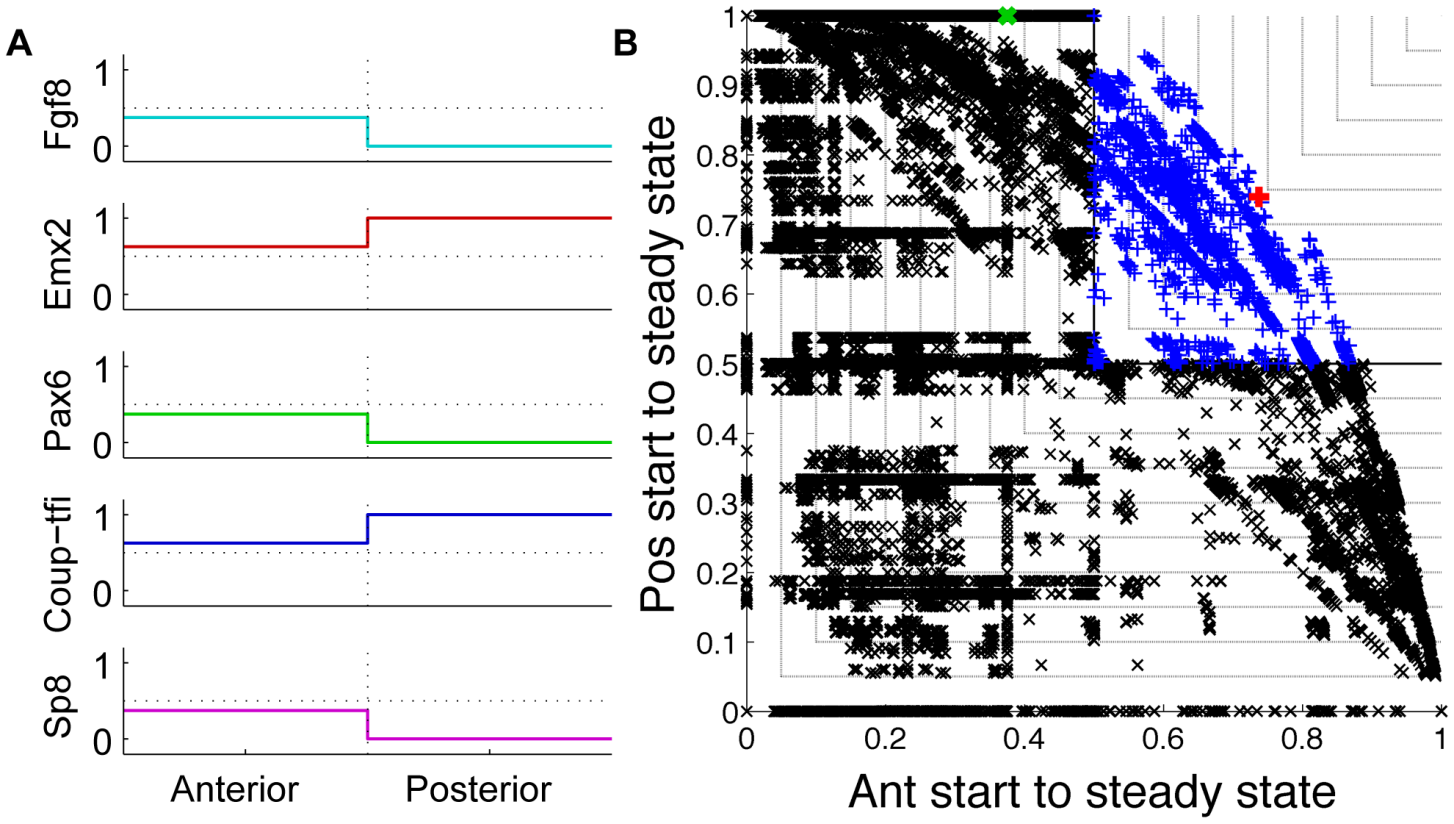
Performance of a previously hypothesised network, and all possible networks. (**A**) The average expression in the anterior and posterior compartments in the experimentally hypothesised network in Figure 2. This network does not satisfy our criteria for reliable performance because the gradients do not span 0.5. (**B**) Each network followed the desired anterior state trajectory and the desired posterior state trajectory with a fixed probability plotted on the two axes of this graph. We defined good networks as those with a greater than 50% chance of following both the desired anterior trajectory of states as well as the desired posterior trajectory of states. These networks lie in the upper, right quandrant of this graph (blue plusses). All other networks (black crosses) did not satisfy our criteria for reliably reproducing the experimentally observed patterns of gene expression. The point corresponding to the experimentally hypothesised network in Figure 2 is coloured green. The red plus corresponds to the two best performing networks in Figure 7B and C. The black contour lines are lines of constant network performance.

To give a specific example, we present the dynamics of a regulatory network previously hypothesised based on experimental observations [5], [8], seen in Figure 2A. The network was converted into the set of Boolean logic equations described in Figure 2C. We found that this network had a 100% chance of following the desired trajectory from the posterior starting state to the posterior steady state. In constrast, it had only a 38% chance of following the desired anterior trajectory from the anterior starting state to the anterior steady state. This poor anterior performance arises because of Fgf8 auto-induction and the Fgf8/Sp8 inductive loop, as we will explain later in more detail. While this network produced the correct activity gradients overall, as seen in Figure 3A, it does not satisfy our criteria for doing so reliably because the gradients do not span 0.5; the anterior levels of *Fgf8*, *Pax6* and *Sp8* are too low (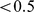), while the anterior levels of *Emx2* and *Coup-tfi* are too high (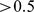). The fact that this network did not reach our criteria for reproducing the experimental observations, even though all interactions have been observed indirectly, shows clearly that intuitions about the dynamics of regulatory networks with feedbacks can be unreliable.

### Only a small percentage of networks satisfied our criteria for reproducing the experimental observations

Of all possible networks, we found that 0.1% of networks (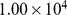) had a greater than 50% chance of proceeding from the anterior starting state to the anterior steady state, as well as a greater than 50% of proceeding from the posterior starting state to the posterior steady state. In a plot of the probability of following the desired anterior trajectory through state space against the probability of following the desired posterior trajectory, these good networks lie in the upper right quadrant (Figure 3B).

**Figure 4:**
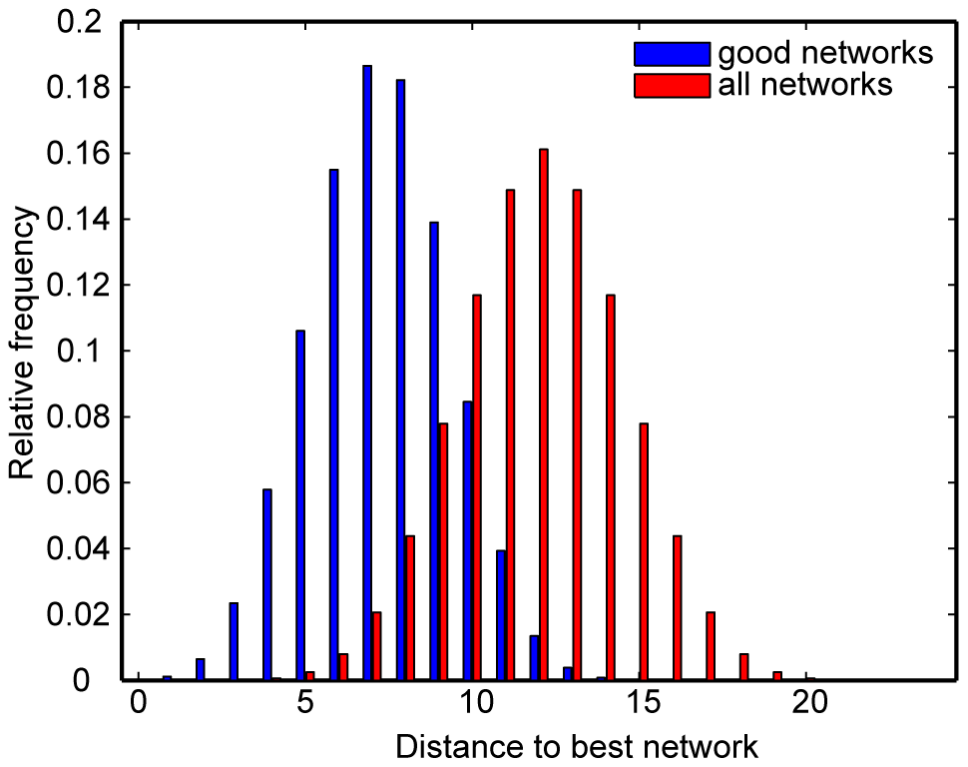
Distribution of the structural difference between the best network and the good networks, as well as the best network and all networks. The distance between two networks is the number of interactions differing between them. The good networks are constrained in their structure so that there is less difference between them and the best network than between all networks and the best network.

To assess the similarity of the structures of the good networks, we calculated the average distance from each of the good networks to the best performing network and compared this to the average distance from all networks (Figure 4). The distance is defined as the number of different interactions [48]. The good networks differ to the best network by an average of 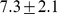 interactions, while all possible networks differ by an average of 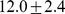 interactions. This indicates that the structures of the good networks are restricted in the space of all possible networks. We then set out to understand which network interactions characterised the good and bad networks.

### There were many combinations of interactions that did *not* appear in networks that performed well

**Figure 5:**
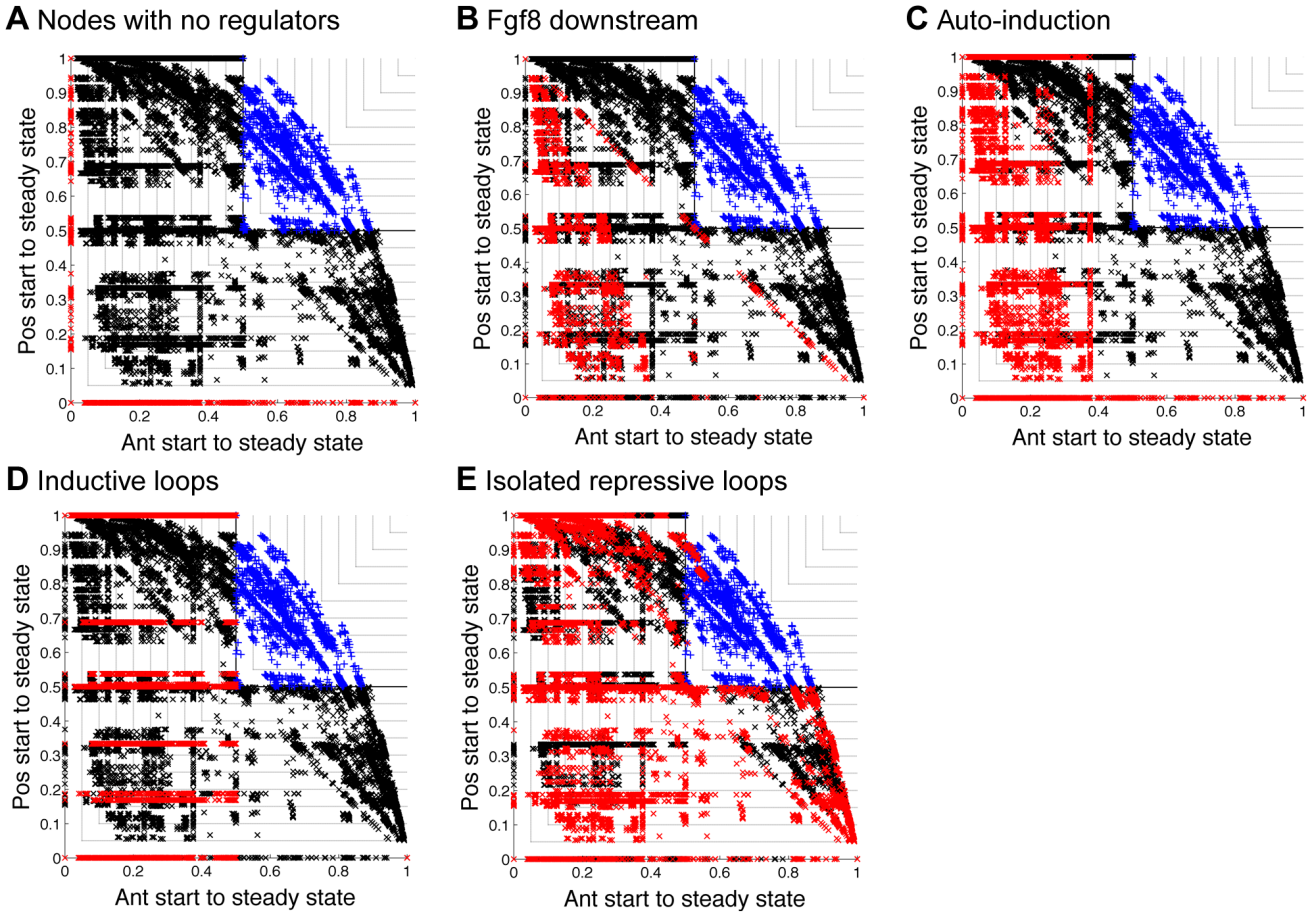
Some combinations of interactions that never appear in good networks. Each panel shows the probability of following the desired anterior state trajectory against the probability of following the desired posterior state trajectory for all 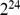 networks that we considered. In each panel, we highlight in red networks that contain a particular combination of interactions. All other bad networks are marked with black crosses, all other good networks are marked with blue pluses. (**A**) In red are networks containing nodes with no regulators. These entered the anterior steady state or posterior steady state but not both. (**B**) In red are networks with *Fgf8* only downstream of the four TFs (*Fgf8* 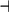
Emx2, *Fgf8* 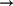
Pax6, *Fgf8* 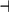
Coup-tfi and *Fgf8* 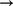
Sp8 all absent). Because the only difference in the starting state between the two compartments was Fgf8 activity, these networks could not enter both the anterior and posterior steady states with 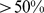 probability. (**C**) Marked in red are networks with auto-induction. Networks with Emx2, Pax6, Coup-tfi or Sp8 auto-induction entered the anterior steady state or the posterior steady state but not both. Networks with Fgf8 auto-induction could reliably enter the posterior steady state but not the anterior steady state. To enter the anterior steady state, they required Sp8 to become and remain active before the state of Fgf8 was updated. Because nodes were updated asynchronously in a random order, this could not occur with 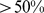 probability. (**D**) In red are networks containing inductive loops. These also could not enter the anterior steady state with 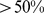 probability by similar reasoning to C. (**E**) In red are networks containing isolated repressive loops (that is, X repressing *Y* was the only regulation of *Y* and Y also repressed X). These also could not reproduce the average gradients observed experimentally.

Careful examination of the interactions present and absent in the good networks allowed us to identify several combinations of interactions that were never present in good networks. Networks containing nodes with no regulators obviously performed poorly. Figure 5A shows their position on the plot of probability of following the desired anterior trajectory though state space against the desired posterior trajectory. In a similar vein, networks where Fgf8 was not upstream of at least one of the four TFs also performed poorly (Figure 5B), because the starting states of the two compartments only differed in Fgf8 activity. In addition, networks with auto-inductive interactions all performed poorly (Figure 5C). This occurs because any node with auto-induction is either locked into its initial state or becomes inactive if it has other regulatory requirements that are not satisfied. Consequently, the desired trajectories cannot occur with a greater than 50% probability in both compartments. By similar reasoning, nodes with inductive loops also performed poorly (Figure 5D), as do networks with isolated repressive loops (Figure 5E).

**Figure 6:**
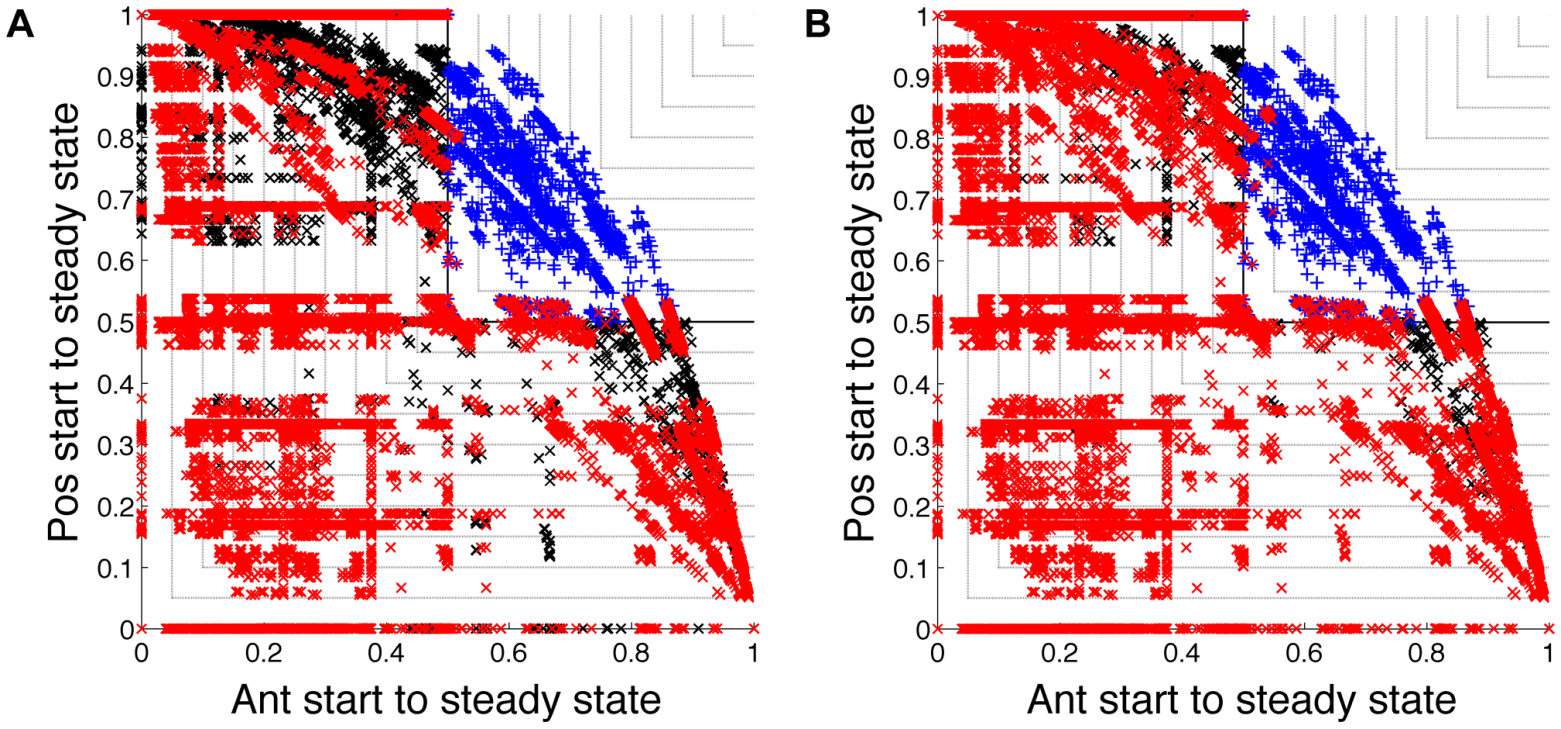
Some higher order combinations of interactions rarely appear in good networks. Each panel shows the probability of following the desired anterior state trajectory against the probability of following the desired posterior state trajectory for all 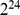 networks that we considered. In each panel, we highlight in red networks that contain particular combinations of interactions. All other bad networks are marked with black crosses, all other good networks are marked with blue pluses. (**A**) In red are networks containing any of the combinations of three interactions listed in Table S1. (**B**) In red are networks containing any of the combinations of four interactions that we found caused networks to perform badly.

We also identified several higher order combinations of interactions that rarely appeared in networks that could produce the average expression gradients observed experimentally. For these higher order combinations, we could not deduce an intuitive explanation for why they caused networks to perform poorly. Some of these combinations are listed in Table S1. Removal of networks containing these interactions further narrowed the space of possible networks as seen in Figure 6. In total, the criteria outlined so far reject 99.96% of the networks investigated, leaving 6980 networks.

### Some interactions were more likely than others to occur in the networks that performed well

By analysing the remaining networks, we identified certain interactions that were more likely than others. Of the remaining networks, 84% (5849 of 6980), satisfied our criteria for reliably following the desired trajectories to produce the average expression gradients observed experimentally. Surprisingly, among these good networks, no single interaction was universally present or absent, except those already identified as deterimental (Table 1, third column). In fact, among the remaining, good networks, all interactions occurred at about the frequency expected from all the remaining networks (Table 1, third column compared to second column). In general, the repressive interactions were more likely than inductive ones. The interactions Fgf8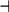 *Emx2* and Fgf8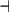 *Coup-tfi* were the most likely interactions, occuring in 80% of all remaining networks that performed well. Next, Emx2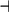 *Sp8* and Coup-tfi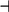 *Sp8* occurred in 66% of good networks. The interactions Pax6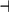 *Emx2* and Pax6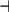 *Coup-tfi* occurred in 55% of all good networks, while Emx2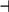 *Pax6* and Coup-tfi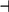 *Pax6* occurred in 54% of all good networks.

**Table 1:** Probability of interactions being present in all remaining networks, compared to remaining networks that perform well.

Interaction	P(present) in all remaining networks (%)	P(present) in all remaining good networks (%)
F 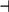 *E*	80	80
F 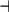 *C*	80	80
E 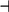 *S*	66	66
C 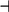 *S*	66	66
P 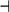 *E*	55	55
P 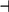 *C*	55	55
E 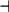 *P*	54	54
C 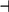 *P*	54	54
S 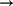 *P*	53	51
F 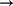 *P*	44	46
E 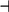 *F*	42	43
C 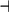 *F*	42	43
S 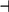 *E*	36	39
S 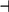 *C*	36	39
C 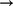 *E*	29	31
E 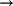 *C*	29	31
P 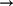 *F*	23	18
P 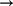 *S*	13	15
F 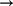 *F*	0	0
E 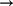 *E*	0	0
P 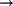 *P*	0	0
C 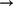 *C*	0	0
S 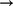 *S*	0	0
F 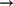 *S*	0	0

All interactions in the good networks occur at about the expected frequency, which means that it is difficult to identify any further combinations of interactions that ensured networks performed badly or well. Despite this, we can still use the probabilities of individual interactions among the remaining good networks as an indicator of which interactions are likely and unlikely to occur in the gene network regulating cortical arealisation.

**Figure 7:**
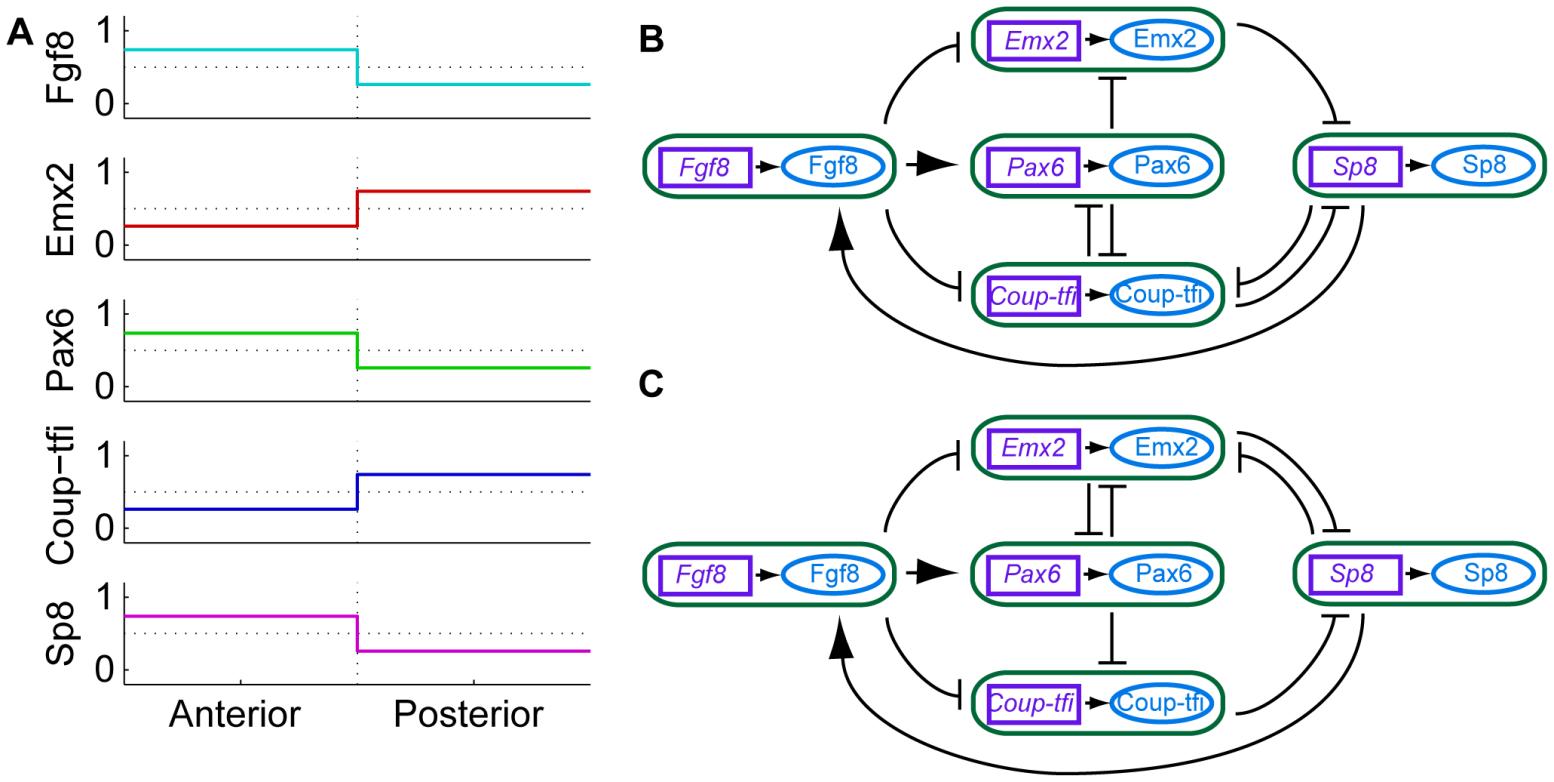
Best performing networks. (**A**) In the best performing networks, the average activity of the genes and proteins of interest in the anterior and posterior compartments formed gradients in the same direction as those observed in mouse (cf Figure 1C). (**B**) The structure of the two best networks. The purple boxes with names in italics represent genes and the blue ellipses with names in upright text represent proteins. Each of the *gene* 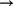
protein interactions has been condensed into a green box to simplify the diagram and avoid intersecting edges. Each edge between the rounded green boxes indicates how the protein in the source box regulates the gene in the target box. The two best networks performed equally well. However, some other networks with quite different structures also performed *nearly as* well.

Though many different networks performed well, we now discuss the best performing networks as an illustrative example. The two best performing networks both followed the desired anterior trajectory 74% of the time and the desired posterior trajectory 74% of the time. They are marked in red in Figure 3B and reliably produced the average expression gradients observed experimentally (Figure 7A cf. Figure 1C). Figure 7B and C show the structures of these two networks. Note that the six most likely interactions from the third column of Table 1 are present in these networks, as well as several less common interactions. However, many networks with similar structures also produced the correct average expression gradients, while some with quite different structures did too. Thus, although the networks that reproduced the experimentally observed expression gradients were constrained in structure compared to all possible structures, there was still a remarkable diversity in these networks.

**Figure 8:**
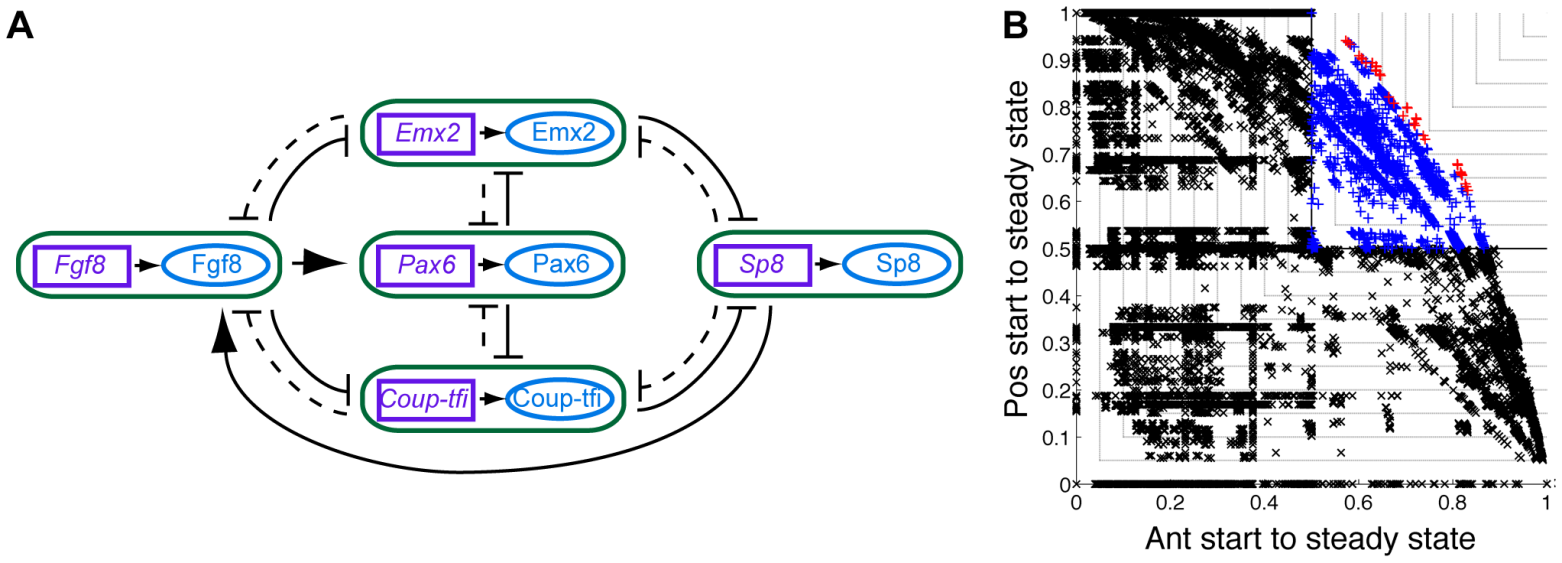
A selection of networks that produced the correct average expression gradients and have common structural elements. (**A**) The structure of the networks. The purple boxes with names in italics represent genes and the blue ellipses with names in upright text represent proteins. Each of the *gene* 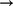
protein interactions has been condensed into a green box to simplify the diagram and avoid intersecting edges. Each edge between the rounded green boxes indicates how the protein in the source box regulates the gene in the target box. The solid lines indicate interactions that must be present while the dashed lines indicate interactions that can be present or absent. The 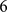 dashed interactions means that this diagram represents 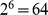 different networks. (**B**) The performance of these 64 networks (red pluses) on a plot of probability of following the desired anterior state trajectory against the probability of following the desired posterior state trajectory. All other good networks are marked with blue pluses, all bad networks with black crosses.

In general, repressive interactions were more prevalent in the networks that performed well than inductive interactions. This is evident in the probabilities of each interaction being present (Table 1, third column), as well as a set of networks that performed similarly to the best performing network, that are illustrated in Figure 8A. In these 64 networks, the six most common interactions in the good networks were all required to be present. All other repressive interactions, which created reciprocal repressive loops, could be present or absent without greatly affecting network performance. The only inductive interaction appearing in this set of networks was Fgf8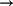 *Pax6*, and it was present in all 64 networks. All inductive interactions between the four TFs were required to be absent along with Pax6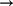 *Fgf8*, all auto-inductive loops and Fgf8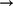 *Sp8* which created an inductive loop.

## Discussion

### The roles of different genes

Current experimental evidence indicates that the gene network that regulates cortical area development has multiple feedback pathways and consequently, it is difficult to understand intuitively. Using a Boolean logic model, we simulated many different possible networks and identified many structural requirements on the networks to ensure good performance.

Our analysis suggests differing roles for the different genes in the network. We show that *Fgf8* expression at the anterior pole, a putative cortical patterning centre, may be sufficient to drive the correct spatial patterning of the transcription factors *Emx2*, *Pax6*, *Coup-tfi* and *Sp8*, if simple interactions between these transcription factors exist. This is an example of how a transient signal, in this case *Fgf8* expression initiated by external regulators, can be converted into a durable change in the developing brain [49].

In our simplified model, *Emx2* and *Coup-tfi*, which are both expressed in high posterior–low anterior gradients, play the same role in the network. This means that if *Emx2* and *Coup-tfi* are swapped in any network, the dynamics of the network don't change. This is evident in the higher order interactions that rarely appear in good networks, listed in Table S1, as well as the two best performing networks in Figure 7B. In reality, *Coup-tfi* has a sharper anterior-posterior expression gradient than *Emx2* and the two TFs are expressed in opposing gradients along the medial-lateral axis. Experiments suggest that Emx2 promotes posterior area identity while Coup-tfi represses anterior area identity [6]. Therefore, we expect that they are not redundant as our model suggests, but play different roles through differing downstream targets.

### Interactions we predict are likely

Our screen of possible networks identified interactions that we predict are more likely to be present in the arealisation regulatory network than others, and are therefore good experimental targets for further study. In general, we predict that repressive interactions are particularly important in this network. This is consistent with data showing that repressive cascades are important for spatial differentiation in other systems [50], [51]. The interactions we predict are most likely include several interactions that have previously been hypothesised based on experiments. Our analysis predicts that Fgf8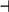 *Emx2* and Fgf8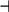 *Coup-tfi* are the most likely direct interactions, consistent with many previous suggestions [20], [21], [27], [52]–[54]. Since Sp8 induces *Fgf8*, repression of *Sp8* by Emx2 has been proposed as a mechanism by which *Fgf8* expression can be contained to the anterior pole [24]. Our analysis predicts that repression of *Sp8* by Emx2 or Coup-tfi, or both, is quite likely. Currently, possible repression of *Sp8* by Coup-tfi has not been discussed in the experimental literature. Reciprocal repression between *Emx2* and *Pax6* has been frequently discussed as potential regulatory interaction [5], [7], [11], [23], [55], [56]; [ but see also 2]. Our analysis predicts that these interactions are approximately equally likely. However, it also predicts that reciprocal repression loops in general are not critical for correct functioning of the network.

### Interactions we predict are unlikely

Our screen of networks also predicts several single interactions and many combinations of interactions that are *unlikely* to occur in the arealisation regulatory network since they usually lead to poor performance. The lack of an intuitive explanation of why some of the combinations of interactions degrade network performance demonstrates the complexity of the network dynamics, and why computational modelling of these networks gives insights not available through intuition. Several of the interactions that we predict are unlikely have previously been hypothesised based on experiments. In particular, Fgf8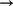 *Sp8* has been proposed by Sahara et al. [24] but our simulations predict that this interaction creates an inductive loop which is detrimental to network performance. The experimental evidence for this interaction is that ectopic expression of *Fgf8* in the telencephalon by in utero electroporation at E11.5 induced ectopic expression of *Sp8* at E13.5 [24]. However, the target tissue contained an active regulatory network that could have indirectly initiated expression of *Sp8* after perturbation by ectopic Fgf8. Direct auto-induction of any of the five genes prevented networks from being able to recreate the experimental expression patterns. Auto-induction of Fgf8 has previously been hypothesised based on experiments implanting Fgf8-coated beads in the chick midbrain [57], limbs [58] and telencephalon [52], but our model predicts the resulting induction of ectopic Fgf8 is unlikely to be direct. It could be occurring indirectly through an active regulatory network perturbed by ectopic Fgf8. For example, in the forebrain, if Emx2 does limit the region of Fgf8 expression by repressing Sp8 inducing Fgf8 (Emx2
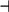
Sp8
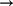
Fgf8, [24]) and *Fgf8* represses Emx2, then ectopic Fgf8 protein could induce the transcription of ectopic *Fgf8* mRNA. A more definitive test of Fgf8 auto-induction would require to addition of ectopic Fgf8 the absence of Sp8 or Emx2. Changes in Fgf8 expression would need to be examined after E12.5 because Fgf8 expression in the forebrain appears to be initiated by regulators outside the network studied here and only maintained by Sp8 [26].

### Relation to other modelling work

To date, we are only aware of one paper modelling cortical arealisation [59]. The model starts with expression gradients of Fgf8, Emx2 and Pax6, which are maintained by regulation of each other. It then goes on to simulate the formation of area-specific thalamocortical connections. In contrast, this paper focuses on modelling pattern generation by the gene regulatory network, and at present does not consider the later process of thalamocortical innervation where less data are available to constrain models.

This paper draws on the ideas used in other Boolean modelling papers in different systems, but a systematic analysis of possible regulatory networks is novel. Although algorithms exist for reverse engineering the Boolean expressions and hence the structure of regulatory networks [60], [61], they require data on the time course of expression levels. For example, Laubenbacher and Stigler [61] tested a reverse engineering algorithm by reconstructing a well-characterised network. They showed that their algorithm only worked well when it used time series data from mutant animals, as well as wild type time series. Currently, these data are unavailable for the system we have investigated for either wild type or mutant animals.

More recently, Wittmann et al. [62] used Boolean modelling to infer regulatory relationships governing the spatial patterning of genes at the midbrain-hindbrain isthmus. They were able to use a spatial, rather than temporal pattern to infer minimal Boolean equations using reverse engineering strategies from digital electronic engineering. Compared to the gene expression patterns at the isthmus however, the arealisation expression patterns are much simpler and consequently do not provide us with many constraints for the reverse engineering algorithm. In any case, for more complex modelling Wittmann et al. added additional interactions hypothesised in the literature. In contrast, our simulation of an experimentally hypothesised network gave a negative result, which led to our systematic screening all possible networks. Our goal was to explore the space of possible networks rather than identify one individual network that could produce the desired results as Wittmann et al. did, when many other sufficient networks likely exist.

Albert and Othmer [63] explored a single well-characterised network (the *Drosophila* segment polarity network) in great detail. Using Boolean analysis, they were able to reproduce mutants and predict novel mutants. Unfortunately, mutants in the arealisation genes exhibit a phenotype of shifted expression gradients of the other genes (see Introduction). These results cannot be reproduced in the two compartment, two level model used in this paper (see Methods, Spatial Compartments for more detail).

An extended model with additional spatial compartments and expression levels, or continuous expression levels would be able to incorporate the mutant data. However, these types of models have many more parameters that cannot be constrained by the qualitative experimental data available in this case. Any systematic exploration or optimisation of parameter space for the large number of possible networks we simulate in this paper would be computationally impossible. For example, an ordinary differential equation model using Michaelis-Menten kinetics has two parameters per interaction (the Hill coefficient and the Michaelis constant), as well as a degradation rate and a constitutive activity rate for each species [35], [43], none of which are constrained by experimental data.

### Communication between cells

In this paper, we have not considered any communication between cells since we model only two compartments at the anterior and posterior poles. However, communication between cells may occur and may be useful. Although we find that many networks can produce the experimentally observed *average* expression patterns, we find that in most cases, each network has more than one accessible steady state from each of the starting states. We speculate that this may be resolved by cell-cell signalling of some kind, most likely by Fgf8, which is known to be a secreted molecule. Such signalling could lock the regulatory networks of nearby communicating cells into the same state. Fgf8 movement by diffusion or some other kind of transport might also generate the smooth gradients of the TFs. An investigation of the effects of Fgf8 diffusion would require a more complex model with more than two discrete expression levels and more than two compartments.

### Conclusions

Overall, our exploration of the dynamical consequences of different structures of the network consistent with experimental data predicts constraints on the structure of the real network. The Boolean approach we used is well suited to the qualitative data currently available, and permitted us to screen a large number of networks. Our results may be used as a starting point for future more realistic models of the gene networks regulating cortical arealisation because the narrowed pool of possible networks may make it feasible to investigate parameter space systematically in a more realistic model with many more free parameters. From an experimental perspective, data on the time course of expression levels at different spatial locations, or even accurate relative protein levels would provide useful constraints to future models. We show here though that even a simple Boolean model reveals logical principals underlying the genetic regulation of cortical arealisation, and may be used to guide future experiments.

## Materials and Methods

### Networks simulated

We examined the dynamics of all possible networks created by the five genes and five proteins of interest in anterior-posterior patterning of cortical areas: *Fgf8*, *Emx2*, *Pax6*, *Coup-tfi* and *Sp8* and their respective proteins. The induction of *Fgf8* by Sp8 has been directly demonstrated [24], and therefore, this interaction was fixed in all the simulated networks. Genes were also always fixed to induce their corresponding protein. To narrow the number of networks considered, other interactions were either inductive (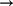) or repressive (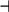), depending on the anterior-posterior expression patterns observed experimentally (shown in Figure 1). For example, because *Emx2* and *Pax6* are expressed in counter gradients, we considered the interactions Emx2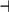 *Pax6* and Pax6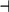 *Emx2* but not the interactions Emx2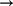 *Pax6* and Pax6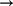 *Emx2*. This gave 24 possible interactions, summarised in Table 2, which have not been directly demonstrated. Hence, we considered all 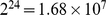 networks formed by different combinations of the possible interactions.

**Table 2:** Summary of all the considered interactions.

Regulator	Target gene or protein									
	*Fgf8*	Fgf8	*Emx2*	Emx2	*Pax6*	Pax6	*Coup-tfi*	Coup-tfi	*Sp8*	Sp8
*Fgf8*		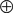								
Fgf8	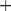		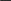		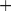		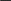		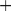	
*Emx2*				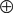						
Emx2	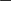		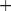		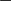		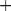		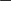	
*Pax6*						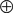				
Pax6	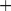		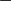		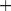		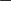		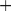	
*Coup-tfi*								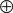		
Coup-tfi	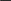		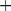		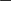		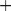		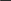	
*Sp8*										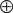
Sp8	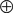		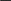		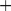		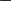		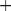	

All possible combinations of these interactions form the space of networks whose dynamics were simulated. Text in italics signifies genes while upright text signifies proteins. A ‘
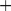
’ indicates an inductive interaction while a ‘
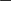
’ indicates a repressive interaction. The table is sparse because we assume that proteins can't regulate proteins and a gene can only regulate its corresponding protein. The circled interactions (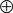) were present in every network because these have been directly demonstrated by experiments. These include each gene producing its respective protein and Sp8 activating *Fgf8*. The other 24 interactions are possible but have not been directly demonstrated. We simulated the dynamics of the 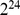 networks formed by all combinations of the possible interactions.

### Converting a network into Boolean logic functions

Each network was turned into a set of Boolean logic functions using the logical operators AND and NOT. Repressive interactions were incorporated with a negation (NOT operator). We assumed that if a gene has multiple regulators, all regulatory conditions must be met, and so we combined their action with a logical conjunction (AND operator). For example, the network in Figure 2A was transformed into the set of Boolean functions in Figure 2C. According to these equations, the state of a gene or protein at time 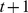 is governed by the state of its regulators at time 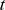. A protein will only be active if its corresponding gene is active at the previous time step, and a gene will only be active if the transcriptional activators of that gene are active at the previous time step and the inhibitors are inactive.

Implicit in these functions are several assumptions [63]: (1) if the regulatory requirements for transcription or translation to occur are satisfied, then the mRNA or protein is synthesised in one time step, (2) mRNA decays within one time step if the necessary regulatory requirements do not continue to be satisfied, and (3) active protein decays within one time step. Albert and Othmer [63] tried relaxing these assumptions and found that it did not change the steady states. We did not consider the OR logical operator, which corresponds to the situation where only one regulatory condition (or a subset of conditions) must be satisfied to set a gene to the active state, or other logical operators. While it would obviously be possible to relax these assumptions, this would cause a large increase in the complexity of the model and a combinatorial explosion in the number of parameters to investigate, making it harder to analyse and derive conclusions from the model.

### Spatial compartments

Since we were interested in anterior-posterior patterning, it was necessary to have a spatial dimension in the model. This was incorporated by considering two compartments, one anterior, one posterior. The regulatory networks, and therefore logic functions, operating in the two compartments were the same. The difference between the two compartments was their initial conditions (outlined later). There was no signalling between compartments.

There are several reasons why signalling between compartments was not incorporated into the model. Firstly, there is currently no experimental evidence for long range communication between cells via our molecules of interest. As discussed in the Introduction, Fgf8 is a secreted protein, and it is hypothesised to diffuse, but only its mRNA expression has been characterised. Even if it does diffuse, it is unlikely to be present at high concentration at the posterior pole, represented in our model by the posterior compartment. Gradients of TF mRNA (and presumably protein) must form by some mechanism other than diffusion and here we assume the TFs act on each other independently in each compartment.

Given the lack of signalling between compartments, and the fact that in Boolean models, each gene and protein can only have the state ‘0’ or ‘1’, two compartments with different initial conditions were sufficient to completely explore the system. Additional compartments between the anterior and posterior extremes would have to start with the same initial conditions as either the anterior or posterior compartment. Without communication between compartments, these hypothetical additional interior compartments would follow the same dynamics as an exterior compartment with the same starting state. Hence, they would not provide any extra information to constrain the structures of the arealisation network.

A consequence of this two-level, two-compartment model is that we cannot simulate the mutant phenotypes of shifting expression gradients (see Figure 2B for references). In the current model, an expression gradient is represented by a protein in the active state in one compartment and in the inactive state in the other compartment. The only other possible expression patterns are active–active or inactive–inactive, which are not shifted gradients.

### Initial states and desired steady states of each compartment

The difference between the two compartments was their initial state, and we were interested in which steady state they each ended up in given the different initial states. We describe the state of the system with a ten-tuple of 1's and 0's representing the state of the network nodes [*Fgf8*, Fgf8, *Emx2*, Emx2, *Pax6*, Pax6, *Coup-tfi*, Coup-tfi, *Sp8*, Sp8]. For example, the state [1,1,0,0,0,0,0,0,0,0] denotes *Fgf8* gene and protein are active, while all other genes and proteins are inactive. This corresponds to the starting state in the anterior compartment at around E8, where *Fgf8* expression is thought to be initiated via a mechanism external to the regulatory network we are modelling [5], [24], [26]. We assume that the expression of the other four genes is controlled by the regulatory network we are modelling. In the posterior compartment, we assume that the expression of all five genes is controlled by the modelled regulatory network and so this compartment starts in the state [0,0,0,0,0,0,0,0,0,0]. The Boolean versions of the desired anterior and posterior steady states are seen in Figure 1C and are given in tuple notation as [1,1,0,0,1,1,0,0,1,1] for the anterior compartment and [0,0,1,1,0,0,1,1,0,0] for the posterior compartment. We were interested in networks which flowed from the anterior starting state [1,1,0,0,0,0,0,0,0,0] to the anterior steady state [1,1,0,0,1,1,0,0,1,1], as well as from the posterior starting state [0,0,0,0,0,0,0,0,0,0] to the posterior steady state [0,0,1,1,0,0,1,1,0,0].

### Creating and analysing state tables

The binary tuple representation of states emphasises the fact that Boolean networks are finite state machines whose steady states can be readily determined. Initially, we determined the steady states of each network by creating a state table for each network. This is a list of all possible states of the network ( [0,0,0,0,0,0,0,0,0,0], [0,0,0,0,0,0,0,0,0,1],…, [1,1,1,1,1,1,1,1,1,1]) and the corresponding next state when the Boolean rules were applied. Steady states were those that did not change under the Boolean rules. Unfortunately however, this analysis could not reveal which networks proceeded along the desired trajectories through state space. Trajectories can be traced in state tables, but such trajectories assume that all nodes update synchronously so that trajectories are deterministic. Synchronous updating has been used previously in Boolean modelling [63] but synchronous trajectories frequently end up in artefactual cyclic attractors [35], [49], [64]. In reality, it is highly unlikely that multiple species in a network would change their state at exactly the same time. Rather, these systems are stochastic, with nodes updated asynchronously, and this has consequences for the dynamics of the system.

### Simulating the networks with asynchronous updating

Assuming fully asynchronous updating of nodes enabled the use of the Markov chain formalism to describe and analyse the network dynamics [65]. The transition matrix 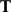 of a Markov chain contains the probability of transition from each state to other states in state space. We used the deterministic state table described above to calculate the transition matrix, 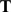, of each network, assuming that each individual node changed state with equal probability. For example, a deterministic transition from [1,1,0,0,0,0,0,0,0,0] to [1,1,0,0,1,1,0,0,0,0] translated to a stochastic transition to state [1,1,0,0,1,0,0,0,0,0] or [1,1,0,0,0,1,0,0,0,0], each with a 50% probability. We found that most networks formed reducible Markov chains, with more than one steady state, each a part of a closed class of states. In general, the anterior and posterior starting states were transient states that could end up at more than one steady state. The probability of ending up at different steady states from a transient state could be calculated analytically [65] or by performing the simple computation:
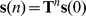
(1)where 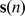 is the distribution of states of the system at time step 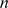. Note that 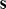 is different to the state tuple notation used so far. Instead, it is a column vector of length 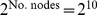. The probability of being in state [0,0,0,0,0,0,0,0,0,0] is given by element 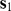, the probability of being in state [0,0,0,0,0,0,0,0,0,1] is given by element 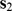, and so on. The two compartments in our model each started in a single state, not a distribution of states. Hence, the anterior starting state [1,1,0,0,0,0,0,0,0,0] corresponded to an 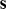-vector with a probability of one at element 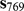 and zero probability elsewhere, and the posterior starting state [0,0,0,0,0,0,0,0,0,0] corresponded to an 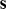-vector with a probability of one in element 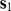 and zero probability elsewhere. The element 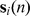 gives the probability of finding the system in state 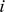 at time step 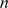. Since our networks always ended up in a distribution of steady states, if 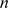 was large enough, the computation of Equation 1 determined the probability of ending up at different steady states from the starting state 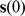. In our analysis, since we knew the steady states of each Markov chain from the state tables, we iteratively calculated 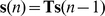 until there was a 99.99% chance of being in the steady states.

In many cases, there was a distribution of steady states. As each compartment represented many cells, the steady state probability distribution could be interpreted as the distribution of states across an inhomogeneous cell population [66]. Hence, we calculated the average amount of each species in a compartment as the sum of steady states of each compartment weighted by the probability of entering that steady state.

### Analysing the similarity between different groups of networks

We quantified the structural difference between two networks as the number of interactions differing between them. We refer to this as the distance between networks because if the network structure is notated as a vector, then our measure of difference between two networks is the Manhattan distance between the two vectors. Because there are 24 possible interactions, the maximum distance between two networks is 24, which occurs if all interactions that are present on one network are absent in the other and vice versa.

### Identifying good and bad networks and good and bad combinations of interactions

We were interested in networks that reliably followed a trajectory from the anterior starting state to the anterior steady state, as well as from the posterior starting state to the posterior steady state. We defined the overall performance index of a network, 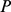, as the minimum of the probability of following the desired anterior trajectory and the probability of following the desired posterior trajectory. If a network proceeded along each of the desired trajectories more than 50% of the time, then this was sufficient to give the average expression gradients observed experimentally. This is equivalent to 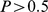. Graphically, we represent the performance of different networks on plots of probability of proceeding from the anterior starting state to the anterior steady state, against probability of proceeding from the posterior starting state to the posterior steady state (for example, see Figure 3B). On these plots, networks that reliably produce the experimentally observed expression gradients (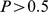) fall in the upper, right quadrant.

Finally, we found combinations of interactions that made a network perform universally poorly or well. We did this by examining the distribution of 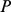 for networks with particular combinations of interactions. We started by looking at 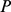 for all networks with each single interaction, compared to without. We then looked at all combinations of interactions being present or absent for all combinations of two, three and four interactions. If all the networks containing a particular combination of interactions had 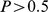, then that set of networks was classified as good. Conversely, if the majority of networks containing a particular combination of interactions had 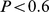, and only a few networks with 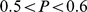, then that set of networks was classified as bad.

## Supporting Information

Table S1 Higher order combinations of interactions that rarely appear in good networks.
(0.04 MB PDF)
