# Supplementary material for: A Boolean Model of the Gene Regulatory Network Underlying Mammalian Cortical Area Development
Source: PLoS Comput Biol. 2010 Sep 16;6(9):e1000936. doi: 10.1371/journal.pcbi.1000936 (PMC2940723; doi:10.1371/journal.pcbi.1000936)
Supplement: Table S1 — Higher order combinations of interactions that rarely appear in good networks. (0.04 MB PDF) [file pcbi.1000936.s001.pdf]

Table S1. Higher order combinations of interactions that rarely appear in good networks.

✓	✓	✓	✓	✓	✗	✓	✗	✗
C→*E*	S⊣*C*	F→*P*	C→*E*	P→*F*	F⊣*C*	C→*E*	F⊣*C*	F→*P*
E→*C*	S⊣*E*	F→*P*	E→*C*	P→*F*	F⊣*E*	E→*C*	F⊣*E*	F→*P*
C→*E*	S⊣*C*	S→*P*	C→*E*	C⊣*F*	F⊣*C*	C→*E*	F⊣*C*	P⊣*C*
E→*C*	S⊣*E*	S→*P*	E→*C*	E⊣*F*	F⊣*E*	E→*C*	F⊣*E*	P⊣*E*
C→*E*	S⊣*C*	P⊣*C*	C→*E*	F⊣*E*	F⊣*C*	C→*E*	F⊣*C*	E⊣*S*
E→*C*	S⊣*E*	P⊣*E*	E→*C*	F⊣*C*	F⊣*E*	E→*C*	F⊣*E*	C⊣*S*
C→*E*	S⊣*C*	C⊣*S*	C→*E*	S⊣*E*	F⊣*C*	S⊣*E*	P→*S*	C⊣*S*
E→*C*	S⊣*E*	E⊣*S*	E→*C*	S⊣*C*	F⊣*E*	S⊣*C*	P→*S*	E⊣*S*
C→*E*	P→*S*	P⊣*C*	C→*E*	C⊣*P*	F⊣*C*	P→*F*	F⊣*E*	F⊣*C*
E→*C*	P→*S*	P⊣*E*	E→*C*	E⊣*P*	F⊣*E*	S→*P*	F⊣*E*	F⊣*C*
C→*E*	P→*S*	C⊣*S*	C→*E*	S→*P*	F⊣*C*	P→*S*	F⊣*E*	F⊣*C*
E→*C*	P→*S*	E⊣*S*	E→*C*	S→*P*	F⊣*E*	P→*S*	E⊣*P*	C⊣*P*
			C→*E*	S⊣*C*	F⊣*C*	F→*P*	E⊣*S*	C⊣*S*
			E→*C*	S⊣*E*	F⊣*E*	S→*P*	E⊣*S*	C⊣*S*
			C→*E*	P→*S*	F⊣*C*			
			E→*C*	P→*S*	F⊣*E*			
			C→*E*	C⊣*S*	F⊣*C*			
			E→*C*	E⊣*S*	F⊣*E*			
			P→*S*	P⊣*E*	F⊣*C*			
			P→*S*	P⊣*C*	F⊣*E*			
			P→*S*	E⊣*S*	F⊣*C*			
			P→*S*	C⊣*S*	F⊣*E*			
			S⊣*E*	S→*P*	F⊣*C*			
			S⊣*C*	S→*P*	F⊣*E*			
			F→*P*	S⊣*E*	C⊣*S*			
			F→*P*	S⊣*C*	E⊣*S*			
			S→*P*	S⊣*E*	C⊣*S*			
			S→*P*	S⊣*C*	E⊣*S*			
			C→*E*	S⊣*C*	P→*S*			
			E→*C*	S⊣*E*	P→*S*			
			P→*S*	P⊣*E*	C⊣*P*			
			P→*S*	P⊣*C*	E⊣*P*			

If any of the combinations of the presence (✓) and absence (✗) of three interactions listed here occurred in a network, then that network was highly unlikely to be able to reproduce the experimentally observed expression patterns. That is, it was likely have a performance index less than 0.5. For some combinations of interactions, a few networks had a performance index $0.5 < P < 0.6$. We also identified many combinations of four interactions that were rarely present in networks that perform well. The interactions are grouped in pairs where all occurrences of *Emx2* and *Coup-tfi* genes are swapped, as well as all occurrences of the respective proteins. This is because these two genes play the same role in the networks in our model so that swapping them doesn't change the network dynamics. See the Discussion for futher detail.
